# Is wax equivalent to tissue in electron conformal therapy planning? A Monte Carlo study of material approximation introduced dose difference

**DOI:** 10.1120/jacmp.v14i1.3991

**Published:** 2013-01-07

**Authors:** Ray R. Zhang, Vladimir Feygelman, Eleanor R. Harris, Nikhil Rao, Eduardo G. Moros, Geoffrey G. Zhang

**Affiliations:** ^1^ School of Medicine and Public Health University of Wisconsin Madison WI; ^2^ Radiation Oncology Moffitt Cancer Center Tampa FL USA

**Keywords:** electron conformal therapy, bolus, Monte Carlo, stopping power ratio

## Abstract

With CT‐based Monte Carlo (MC) dose calculations, material composition is often assigned based on the standard Hounsfield unit ranges. This is known as the density threshold method. In bolus electron conformal therapy (BolusECT), the bolus material, machineable wax, would be assigned as soft tissue and the electron density is assumed equivalent to soft tissue based on its Hounsfield unit. This study investigates the dose errors introduced by this material assignment. BEAMnrc was used to simulate electron beams from a Trilogy accelerator. SPRRZnrc was used to calculate stopping power ratios (SPR) of tissue to wax, $SPR_{wax}^{tissue}$, and tissue to water, $SPR_{water}^{tissue}$, for 6, 9, 12, 15, and 18 MeV electron beams, of which 12 and 15 MeV beams are the most commonly used energies in BolusECT. DOSXYZnrc was applied in dose distribution calculations in a tissue phantom with either flat wax slabs of various thicknesses or a wedge‐shaped bolus on top. Dose distribution for two clinical cases, a chest wall and a head and neck, were compared with the bolus material treated as wax or tissue. The $SPR_{wax}^{tissue}$ values for 12 and 15 MeV beams are between 0.935 and 0.945, while the $SPR_{water}^{tissue}$ values are between 0.990 and 0.991. For a 12 MeV beam, the dose in tissue immediately under the bolus is overestimated by 2.5% for a 3 cm bolus thickness if the wax bolus is treated as tissue. For 15 MeV beams, the error is 1.4%. However, in both clinical cases the differences in the PTV DVH is negligible. Due to stopping power differences, dose differences of up to 2.5% are observed in MC simulations if the bolus material is misassigned as tissue in BolusECT dose calculations. However, for boluses thinner than 2 cm that are more likely encountered in practice, the error is within clinical tolerance.

PACS numbers: 87.55.km, 87.56.ng

## I. INTRODUCTION

Electron beams are commonly used for treatment of shallow tumors.^(^ ^1^ ^)^ To compensate for body surface irregularities and/or to modulate the therapeutic depth, a bolus is often employed.^(^ ^2^ ^–^ ^4^ ^)^ The chest wall^(^ ^4^ ^,^ ^5^ ^)^ and head and neck^(^ ^6^ ^)^ are among the sites treated with this technique.

Required bolus shape depends on beam energy, external body contour, and target volume. Assuming similar linear electron stopping power between the bolus material and tissue and ignoring multiple Coulomb scattering, a simple approach makes the thickness of the bolus along a fan line such that the depth from the surface to the distal edge of the target volume remains constant.^(^ ^5^ ^,^ ^7^ ^)^ More recent computer‐aided bolus design uses a three‐dimensional (3D) pencil beam algorithm for dose calculation and takes into account electron scatter, distance from the virtual source, and loss of side scatter equilibrium.^(^ ^3^ ^,^ ^8^ ^)^ The collision stopping powers and the scattering powers are assigned based solely on the density empirically correlated to the CT number.^(^ ^7^ ^)^

A commercial system using this approach — known as bolus electron conformal therapy (BolusECT) — is available (.decimal Inc, Sanford, FL). A customized bolus which conforms to the patient's surface anatomy is designed during CT‐based treatment planning and then manufactured from machinable wax by a computer‐aided milling machine. As a part of the quality assurance (QA) process, a new CT scan with the bolus in place is acquired, and the dose is recalculated. It should closely match the original plan where the bolus is represented by a region of interest assigned the bolus material density. Our current TPS (Pinnacle v.9.2, Philips Radiation Oncology Systems, Fitchburg, WI) uses a pencil beam algorithm as the electron dose calculation engine. It is known to produce some dose errors when heterogeneities and irregular surface contours are present.^(^ ^8^ ^,^ ^9^ ^)^ Monte Carlo (MC) electron calculations are more accurate under those conditions.^(^ ^10^ ^)^

Any TPS that uses a pencil beam electron calculation algorithm treats different materials as water of varying density. That includes the bolus material. On the other hand, in MC simulations tissue types (chemical compositions) are differentiated. It is often accomplished by the CT number threshold method.^(^ ^11^ ^,^ ^12^ ^)^ Based on the CT number range, body regions are typically binned into air, lung, tissue, and bone. This is a reasonable approximation, as long as the dose is calculated to the media (not to water).^(^ ^13^ ^)^ The CT number of the machinable wax falls into the soft tissue range, and the bolus is treated as such despite the difference in chemical composition. This type of media misassignment can potentially lead to significant dose errors in MC dose calculations.^(^ ^11^ ^)^ In this paper, we investigate how it affects BolusECT dosimetry.

## II. MATERIALS AND METHODS

### A. Wax properties

Cross‐sectional data for wax were generated using PEGS in EGSnrc.^(^ ^14^ ^)^ The wax density is $0.92\;g/{\text{cm}}^{3}$ and the chemical formula is $C_{25}H_{52}$, with about 14.8% of hydrogen by mass (MachinableWax Inc, Lake Ann, MI). In comparison, human soft tissue contains about 10.6% of hydrogen and over 55% of oxygen.^(^ ^12^ ^)^ Stopping power ratios (SPR) of tissue to wax, $SPR_{wax}^{tissue}$, were calculated for various electron beam energies using SPRRZnrc, another user code of EGSnrc, and compared with the SPR values of tissue to water, $SPR_{water}^{tissue}$. Depth dose in a homogeneous wax phantom, normalized to 1 cGy/MU at the depth of maximum dose ($d_{\max}$) in tissue, was compared with those in water and tissue of the same density. Depth dose curves in tissue with wax slabs of various thicknesses were also compared.

### B. Monte Carlo simulations

An EGSnrc based Monte Carlo program BEAMnrc^(^ ^15^ ^)^ was used for a Trilogy (Varian Medical Systems, Palo Alto, CA) linear accelerator simulation. Percentage depth dose (PDD) curves and profiles of various field sizes were calculated and matched with measurements to within 2% in the high dose and 1 mm in the penumbra regions, respectively. Another EGSnrc‐based code, DOSXYZnrc,^(^ ^16^ ^)^ was used for the phantom and patient dose calculations.

A wedge‐shaped bolus was simulated to study the dose distribution difference in tissue with the wedge material treated as wax or as tissue. The field size was $20\times 20\;{\text{cm}}^{2}$. The heel of the wedge was 5 cm thick (not including the walls), simulated by 25 0.2 cm steps. A homogeneous tissue phantom was positioned immediately under the wedge. The scoring dose grid size inside the phantom was $0.8\times 0.8\times 0.3\;{\text{cm}}^{3}$, with a voxel matrix of 25×25×28. Typical BolusECT electron energies of 12 and 15 MeV were studied

A computer program written in C was used to assign a specific material to a contoured region on a CT dataset for MC simulations.

Two relevant clinical cases were simulated: a left chest wall and a head and neck, comparing the compensator bolus being treated in dose calculations as tissue or wax. The PTV DVHs were compared for these different bolus material assignments.

In the chest wall case, the BolusECT was used for a boost treatment of 8 fractions at 2 Gy per fraction to cover 95% of the PTV. A total of 268 monitor units (MU) per fraction using a 12 MeV beam were delivered. The applicator size was $25\times 25\;{\text{cm}}^{2}$ and a custom cerrobend cutout was used to conform the beam to the PTV.

The same 12 MeV beam was also used for the head‐and‐neck case. The prescription was that 95% of the 64 Gy (32 fractions at 2 Gy per fraction) was to cover 98% of the PTV. A total of 244 MU per fraction were delivered with a $10\times 10\;{\text{cm}}^{2}$ applicator containing a custom cutout.

The beam output is set at 1 cGy/MU at $d_{\max}$ in water for a $10\times 10\;{\text{cm}}^{2}$ field at source‐to‐surface distance (SSD) of 100 cm. Monte Carlo simulations were performed for the 12 MeV beam in this geometry, and the maximum dose on the central axis in Gy/particle was equated to the normalization dose of 1 cGy/MU. All MC calculations for the clinical cases were normalized to this dose value to get the absolute doses.

## III. RESULTS

### A. Stopping power ratios and percentage depth doses

**Figure 1:**
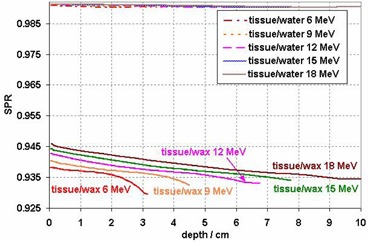
Stopping power ratio (SPR) curves with track ends of tissue/wax for 6, 9, 12, 15, and 18 MeV realistic beams. Tissue/water SPR curves for all the energies are also shown for reference. The 1 SD error bars are too small to be shown in the figure.

Figure 1 shows $SPR_{water}^{tissue}$ along the central axes of the 6, 9, 12, 15, and 18 MeV realistic electron $10\times 10\;{\text{cm}}^{2}$ beams from a Trilogy linear accelerator, with $SPR_{water}^{tissue}$ shown as reference. While $SPR_{water}^{tissue}$ does not show appreciable energy dependency between the beams, there are small but noticeable differences between the $SPR_{wax}^{tissue}$ curves for these energies (Fig. 1).

While $SPR_{water}^{tissue}$ is greater than 0.99, the $SPR_{wax}^{tissue}$ falls approximately between 0.933 and 0.945 for the 12 and 15 MeV beams. In the other words, the stopping powers of tissue and water differ by less than 1%, but the stopping power of wax is higher than that of tissue by 6%−7% (Fig. 1).

**Figure 2:**
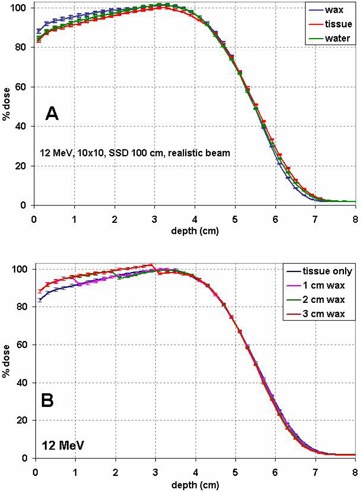
Percentage depth dose (PDD) curves of a 12 MeV beam normalized to the maximum dose in tissue for (A) tissue, water or wax, and (B) heterogeneous phantom consisting of wax slabs of variable thickness on top of tissue.

As a result of this higher stopping power, the central axis depth dose in wax demonstrates higher doses in the buildup and plateau regions and a shorter practical range compared to tissue and water. Figure 2(A) illustrates this difference for a 12 MeV $10\times 10\;{\text{cm}}^{2}$ beam. The physical densities of wax, water, and tissue used in these PDD calculations were all set to the same $0.92\;g/{\text{cm}}^{3}$. The practical range, determined by the depth of the point where the tangent at the inflection point of the falloff portion of the curve intersects the bremsstrahlung background,^(^ ^17^ ^)^ is about 6.5 cm in wax compared to about 6.7 cm in tissue for the 12 MeV beam. The PDD curve in water represents the depth dose in tissue much better than the corresponding curve in wax does. Figure 2(B) demonstrates the PDD for a 12 MeV beam with various thicknesses of wax slabs on top of the tissue phantom. All depth dose curves in Fig. 2 are normalized to the maximum dose in tissue along central axis. Assigning tissue as the bolus material would underestimate the absorbed dose inside the bolus and overestimate the dose behind the bolus. For the 12 MeV beam with a 3 cm wax slab, the dose in tissue just under wax is overestimated by 2.5%±0.7% (1 SD) if wax is treated as tissue. With 2 cm of wax, it is 1.6%±0.8%. The difference is negligible under 1 cm of bolus. The dose difference slowly decreases with depth beyond the wax slab and at 1 cm from the interface, the difference becomes negligible.

**Figure 3:**
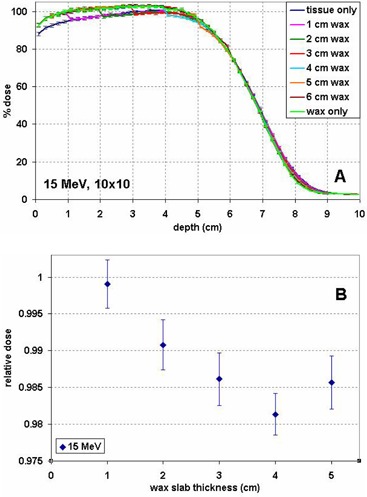
Percentage depth dose (PDD) curves for a 15 MeV beam (A), normalized to the maximum dose in tissue, for wax slabs of various thicknesses on top of tissue and in tissue or wax only. Relative dose in tissue at the wax–tissue interface (B) for wax slabs of various thicknesses, normalized to the dose at the corresponding depth in a homogeneous tissue phantom.

Figure 3(A) shows the PDDs for the $10\times 10\;{\text{cm}}^{2}$ 15 MeV beam with wax slabs of various thicknesses on top of the tissue phantom. The PDD curves are normalized to the maximum dose in the tissue phantom. The same physical density of $0.92\;g/{\text{cm}}^{3}$ is used for wax and tissue in the PDD calculations. The dose difference within the wax slabs is more pronounced than in distal tissue. The practical range difference between tissue and wax can be estimated from this figure at about 8.2 cm in wax vs. 8.4 cm in tissue. Figure 3(B) shows the relative doses in tissue at the wax–tissue interface with wax slabs of various thicknesses. For the 15 MeV beam with a 4 cm wax bolus, the dose in tissue just under the wax is overestimated by 1.9%±0.3% if wax is treated as tissue. The error is 1.4%±0.4% with 3 cm of wax. Table 1 summarizes the calculated percentage dose overestimation of various thicknesses of wax when wax is treated as soft tissue for various energies.

**Table 1:** The percentage of dose overestimation under various thicknesses of wax when wax is treated as soft tissue for various energies of electron beams. Thicknesses effective for treatment depth modulation are listed for each energy.

*Wax Thickness (cm)*	*0.5*	*1*	*2*	*3*	*4*	*5*
6 MeV	1.4%±1.1%	3.2%±0.8%				
9 MeV	0.6%±1.1%	1.3%±0.9%	3.1%±0.5%			
12 MeV	0.4%±1.3%	0.2%±1.2%	1.6%±0.8%	2.5%±0.7%		
15 MeV	‐0.1%±0.4%	0.1%±0.3%	0.9%±0.3%	1.4%±0.4%	1.9%±0.3%	
18 MeV	‐0.2%±1.1%	0.6%±1.3%	0.3%±1.0%	1.1%±0.8%	1.7%±0.7%	3.1%±0.7%

### B. The wedge bolus study

**Figure 4:**
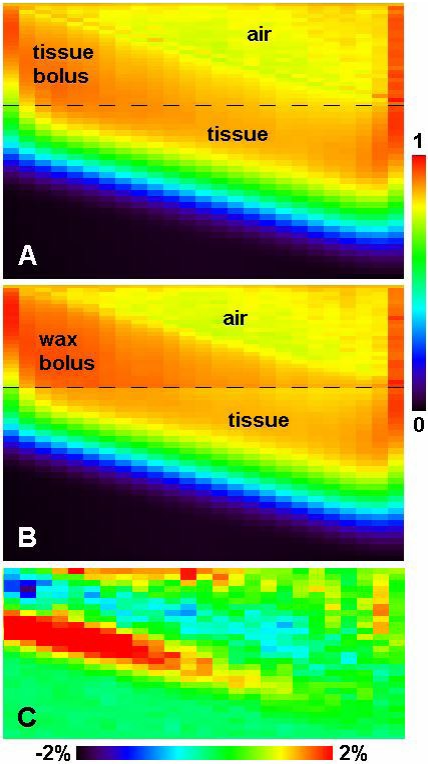
The sagittal view of the central cut through the dose distributions produced by the 15 MeV beam on the wedge bolus and tissue phantom, (A) when the wedge is simulated as tissue, (B) when the wedge is simulated as wax, and (C) the dose difference of A‐B in the homogeneous tissue phantom (wedge and air regions not included). The percentage difference is normalized to the global maximum. All doses are normalized to the global maximum dose in the wax wedge setup. The bolus–tissue phantom interface is illustrated by the dark long dashed lines in (A) and (B).

Figure 4 shows a sagittal view of the central cut through the dose distributions produced by a 15 MeV beam on the tissue phantom with a wedge bolus on top. As expected, when the bolus is simulated as tissue, the dose distribution is continuous between the bolus and the phantom (Fig. 4(A)), while when the bolus is treated as wax, lower dose in tissue at the wax–tissue interface can be clearly observed (Fig. 4(B)). At the bolus–phantom interface, the difference of the two dose distributions reaches its maximum under the heel. For a 15 MeV beam, at 4 cm bolus thickness, the dose in tissue immediate under the wax is overestimated by about 2% of the global maximum dose if the wax wedge is treated as tissue (Fig. 4(C)). Under the toe of the wedge, this overestimation is negligible. The large positive differences shown in Fig. 4(C) are due to the electron beam practical range difference which manifests itself under the heel.

The same wedge bolus simulation for a 12 MeV beam shows slightly larger overestimation, about 2.5% under the bolus region 3 cm thick, which is consistent with the wax slab simulations shown in Fig. 2(B).

### C. Clinical cases

**Figure 5:**
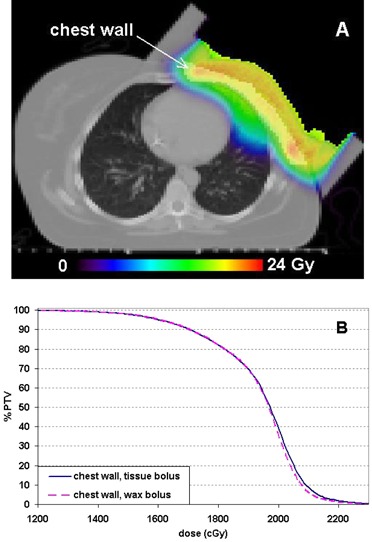
A left chest wall treatment case simulated in this study: (A) the MC calculated dose distribution with the bolus assigned as wax with the chest wall volume highlighted; and (B) PTV DVH comparison between the calculations with the bolus simulated as tissue or wax.

The left chest wall case was simulated with the bolus being treated as either tissue or wax. The PTV consisted of a chest wall and a mastectomy scar, as indicated in Fig. 5. The difference between the DVHs corresponding to the wax or tissue bolus materials was small (Fig. 5(B)). The reason for this small difference is that the bolus is relatively thin throughout the PTV region (≤2 cm). This is representative of a typical chest wall treatment.

**Figure 6:**
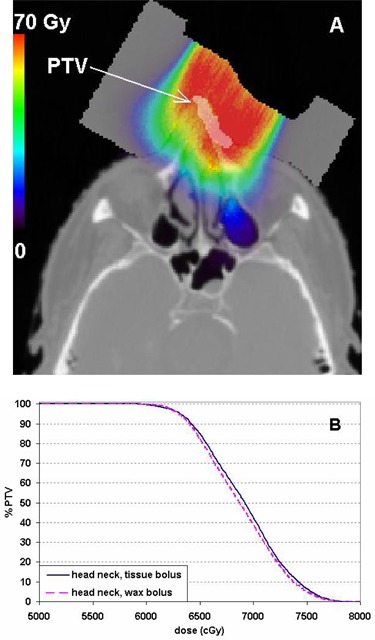
The MC calculated dose distribution (A) with the bolus assigned as wax on a head‐and‐neck case with the PTV highlighted. PTV DVH comparison (B) between the calculations with the bolus simulated as tissue or wax.

In the simulated head and neck case (Fig. 6), the PTV DVH also showed little difference between the wax and tissue bolus material assignments (Fig. 6(B)). The maximum dose difference between the DVH curves was about 50 cGy, or 0.7%, at the DVH point, corresponding to 50% of the PTV volume.

## IV. DISCUSSION

The collisional stopping power, which is responsible for the electron beam dose deposition in the media, is the result of Coulomb interactions. Thus the mass stopping power is proportional to the electron density of the media, which is in turn proportional to the Z/A ratio of the material, where Z is the atomic number and A is the mass number. For all elements, except hydrogen, the Z/A ratio is close to one‐half, with slightly lower values for the high Z materials. For hydrogen, Z/A=1. Thus, for materials with high hydrogen content, the collisional mass stopping power would be higher. The higher hydrogen concentration in machineable wax (14.8% by mass) compared to human tissue (10.6%) is the reason for the higher electron stopping power in wax. The hydrogen concentration in water is 11.1%, which explains why water is a good approximation of tissue dosimetrically, and why $SPR_{water}^{tissue}$ is slightly less than unity.

As the $SPR_{wax}^{tissue}$ values are always smaller than unity, assigning tissue as the bolus material in MC calculations would underestimate the absorbed dose inside the bolus and overestimate the dose behind the bolus. The most prominent dose difference is inside the bolus, which is fortunately not of clinical concern.

As the function of the bolus is to modulate the treatment depth, the useful range of the bolus thickness is 0 to 3.5 cm for the 12 MeV beam and 0 to 4 cm for the 15 MeV beam. Because of the relatively large modulation ranges, these two energies are most commonly used clinically in BolusECT. Within these ranges, the dose errors introduced by misassignment of the bolus material can reach 2.5% and 1.9% of the global maximum dose for 12 and 15 MeV energies, respectively. For electron beams of lower energies, due to smaller modulatable ranges, they are usually not used in BolusECT clinically. For higher energies, due to the deeper penetration thus higher radiation dose to deeper critical structures, their clinical usage in BolusECT is rare, too.

The largest dose errors occur close to the end of the practical range under the thick portion of the bolus (Fig. 4(C)). Clinically, this dose overestimation would primarily affect the organ at risk (OAR) distal to the PTV, if any. This could result in a more conservative dose to the OAR in Monte Carlo treatment planning, which is not harmful to the patient.

To achieve maximum dosimetric accuracy in BolusECT, two issues must be addressed. The first one is the general accuracy of the dose calculation engine, used for both bolus design and final dose calculation, in the presence of nonflat surfaces and patient heterogeneities. This can be relatively easily addressed by using a more accurate electron dose calculation algorithm such as Monte Carlo. However the use of MC calculations results in a second potential issue, namely dose errors resulting from the bolus material, wax, being automatically assumed by the TPS as dosimetrically equivalent to soft tissue. The ability to contour the specific regions of the CT dataset and manually assign the right materials to those regions in MC dose calculation should solve this second potential problem and thus enhance the accuracy of BolusECT planning, providing that the commercial TPS makes the right materials available in the MC system. It is not difficult to generate the cross‐sectional data for known materials using the BEAMnrc package. To make these data available in a commercial TPS may be difficult for the TPS users.

When pencil beam algorithms are used in electron beam planning, by using the depth scaling factor,^(^ ^18^ ^)^ the electron beam practical range can be corrected for the thick wax regions. But the dose in tissue distal the bolus cannot be corrected by this factor alone. The electron fluence scaling factor^(^ ^19^ ^)^ can be applied to correct the dose inside the wax bolus, which is not of clinical concern for patient. This factor again does not help in dose correction in tissue behind the bolus. However, as demonstrated by the two clinical cases, even with the bolus material misassigned as tissue and without any correction applied, the dose difference in the target volume is within clinical tolerance.

## V. CONCLUSIONS

In Monte Carlo dose calculation for bolus electron conformal therapy (BolusECT), dosimetric errors may be introduced in the patient if the bolus material (wax) is automatically (mis)assigned as tissue, especially at locations behind the thick portion of the bolus. Within the clinically relevant bolus thickness range, the dose error can reach up to 2.5% of the global maximum. However, for boluses thinner than 2 cm that are more likely encountered in practice, the error is within clinical tolerance.

## References

[1] Hogstrom KR , Almond PR . Review of electron beam therapy physics. Phys Med Biol. 2006;51(13):R455–R489. 16790918 10.1088/0031-9155/51/13/R25

[2] Kudchadker RJ , Hogstrom KR , Garden AS , Garden AS , McNeese MD , Boyd RA , Antolak JA . Electron conformal radiotherapy using bolus and intensity modulation. Int J Radiat Oncol Biol Phys. 2002;53(4):1023–37. 12095572 10.1016/s0360-3016(02)02811-0

[3] Low DA , Starkschall G , Bujnowski SW , Wang LL , Hogstrom KR . Electron bolus design for radiotherapy treatment planning: Bolus design algorithms. Med Phys. 1992;19(1):115–24. 1620038 10.1118/1.596885

[4] Perkins GH , McNeese MD , Antolak JA , Buchholz TA , Strom EA , Hogstrom KR . A custom three‐dimensional electron bolus technique for optimization of postmastectomy irradiation. Int J Radiat Oncol Biol Phys. 2001;51(4):1142–51. 11704339 10.1016/s0360-3016(01)01744-8

[5] Archambeau JO , Forell B , Doria R , Findley DO , Jurisch R , Jackson R . Use of variable thickness bolus to control electron beam penetration in chest wall irradiation. Int J Radiat Oncol Biol Phys. 1981;7(6):835–42. 6793547 10.1016/0360-3016(81)90483-1

[6] Kudchadker RJ , Antolak JA , Morrison WH , Wong PF , Hogstrom KR . Utilization of custom electron bolus in head and neck radiotherapy. J Appl Clin Med Phys. 2003;4(4):321–33. 14604422 10.1120/jacmp.v4i4.2503 PMC5724465

[7] Hogstrom KR , Mills MD , Almond PR . Electron beam dose calculations. Phys Med Biol. 1981;26(3):445–59. 6787621 10.1088/0031-9155/26/3/008

[8] Boyd RA , Hogstrom KR , Starkschall G . Electron pencil‐beam redefinition algorithm dose calculations in the presence of heterogeneities. Med Phys. 2001;28(10):2096–104. 11695771 10.1118/1.1406521

[9] Fippel M , Kawrakow I , Friedrich K . Electron beam dose calculations with the VMC algorithm and the verification data of the NCI working group. Phys Med Biol. 1997;42(3):501–20. 9080532 10.1088/0031-9155/42/3/005

[10] Fragoso M , Pillai S , Solberg TD , Chatty LI . Experimental verification and clinical implementation of a commercial Monte Carlo electron beam dose calculation algorithm. Med Phys. 2008;35(3):1028–38. 18404938 10.1118/1.2839098

[11] Verhaegen F and Devic S . Sensitivity study for CT image use in Monte Carlo treatment planning. Phys Med Biol. 2005;50(5):937–46. 15798266 10.1088/0031-9155/50/5/016

[12] White DR , Woodard HQ , Hammond SM . Average soft‐tissue and bone models for use in radiation dosimetry. Br J Radiol. 1987;60(717):907–13. 3664185 10.1259/0007-1285-60-717-907

[13] Vanderstraeten B , Chin PW , Fix M , et al. Conversion of CT numbers into tissue parameters for Monte Carlo dose calculations: a multi‐centre study. Phys Med Biol. 2007;52(3):539–62. 17228104 10.1088/0031-9155/52/3/001

[14] Kawrakow I . Accurate condensed history Monte Carlo simulation of electron transport. I. EGSnrc, the new EGS4 version. Med Phys. 2000;27(3):485–98. 10757601 10.1118/1.598917

[15] Rogers DWO , Faddegon BA , Ding GX , Ma CM , We J , Mackie TR . BEAM: a Monte Carlo code to simulate radiotherapy treatment units. Med Phys. 1995;22(5):503–24. 7643786 10.1118/1.597552

[16] Kawrakow I and Walters BRB . Efficient photon beam dose calculations using DOSXYZnrc with BEAMnrc. Med Phys. 2006;33(6):3046–56. 16964882 10.1118/1.2219778

[17] Khan FM , Doppke KP , Hogstrom KR , et al. Clinical electron‐beam dosimetry: report of AAPM Radiation Therapy Committee Task Group No. 25. Med Phys. 1991;18(1):73–109. 1901132 10.1118/1.596695

[18] Ding GX and Rogers DW . Mean energy, energy‐range relationships and depth‐scaling factors for clinical electron beams. Med Phys. 1996;23(3):361–76. 8815379 10.1118/1.597788

[19] Ding GX , Rogers DW , Cygler JE , Mackie TR . Electron fluence correction factors for conversion of dose in plastic to dose in water. Med Phys. 1997;24(2):161–76. 9048356 10.1118/1.597930

